# A Static Area Coverage Algorithm for Heterogeneous AUV Group Based on Biological Competition Mechanism

**DOI:** 10.3389/fbioe.2022.845161

**Published:** 2022-04-13

**Authors:** Xuan Guo, Yuepeng Chen, Dongming Zhao, Guangyu Luo

**Affiliations:** School of Automation, Wuhan University of Technology, Wuhan, China

**Keywords:** Voronoi diagram, biological competition mechanism, load balancing, heterogeneous autonomous underwater vehicle group, centroidal Voronoi tessellation algorithm

## Abstract

Static area coverage of the heterogeneous autonomous underwater vehicle (AUV) group is widely used in many fields. With the use of the centroidal Voronoi tessellation (CVT) algorithm, the coverage problem can be resolved. However, the CVT method, which is optimized with the location evaluation function, cannot consider the heterogeneity of AUVs when the group performs the static area coverage task and will cause a waste of resources. In this paper, considering different AUVs’ task requirements and detection capabilities comprehensively, we propose a coverage control optimization algorithm based on a biological competition mechanism (BCM). By using BCM, the task load of each AUV can be distributed consistently. In addition, we provide strict proof of the consistency of the algorithm based on the Lyapunov method. Simulation results demonstrate that with the proposed algorithm, the location distribution of the heterogeneous AUV group for area coverage is close to the balanced value, and the performance is better than the CVT algorithm for static area coverage.

## 1 Introduction

Interest in the development of the autonomous underwater vehicle (AUV) has grown rapidly over the past few decades. AUV plays an important role in ocean exploration and development, such as deep-sea inspections, long-term surveys, and oceanographic mapping for detecting, locating, and eliminating submarine mines (Hussain et al., 2020; Makavita et al., 2020; Kuhlman et al., 2021). However, it is difficult for a single AUV to complete complex or high-disk tasks with low cost and fewer sensors, especially in the presence of uncertainty, incomplete information, and distributed control. To make up for the inadequacy of a single AUV, the AUV group is used for many marine tasks (Sun et al., 2020; Yan et al., 2020). AUV group faces the problem of area coverage in the process of carrying out tasks such as seafloor sonar array exploration, resource exploration, and ocean hydrographic environment exploration (Antonelli and Antonelli, 2014; Wadoo and Kachroo, 2017; Yan et al., 2019; Xin et al., 2021; Yang et al., 2021). In conditions where the task area environment is unknown, the AUVs scan the entire area by sensors with a specific detection range. The static area coverage problem is the static deployment of the AUVs in the target area for detection according to the needs of the detection task (Zhong and Cassandras, 2008). The primary approach to static area division is the Voronoi diagram method (Cortes et al., 2004). The distribution with the location optimal function as the optimization objective is called the CVT method, which can assign each AUV into the area where the nearest one of the initial points is located, and the density in the area is considered comprehensively to achieve a uniform distribution of the area.

However, with the increasing complexity and the danger of underwater tasks, a single AUV or a homogeneous AUV group is difficult to complete the tasks. In order to improve efficiency of task execution, AUVs with different characteristics and abilities are grouped to perform tasks collaboratively, which is called heterogeneous AUV group (HEAUVs). In HEAUVs, since each AUV has a different task load and often has different ability to complete tasks, there is a problem of task load imbalance. For the static area coverage problem of HEAUVs, AUVs are usually made and produced from different manufacturers. They often carry different types and numbers of sensors and thus have different detection ranges and capabilities. Therefore, the CVT method that relies on the distance evaluation function for allocation results in a waste of resources and cannot optimally solve the static coverage problem of HEAUVs (Cassandras and Li, 2005). The competition of organisms for territory and resources in nature can eventually reach a state of relative equilibrium and realize the rational allocation of resources. This behavior can be called a biological competition mechanism (BCM). BCM provides a solution to the problem of uneven distribution of regions in HEAUVs due to different task requirements and detection capabilities (Buiu and Florea, 2019; Mitrana, 2019; Barbuti et al., 2020). In this paper, BCM is introduced to establish the allocation model of the target area, and the optimal allocation of static regions of HEAUVs is achieved with the optimization goal of balancing the AUV detection capability with the task demand.

Researchers have done a lot of research on static area coverage. Using the Voronoi diagram to divide the task environment into multiple and non-overlapping Voronoi units is introduced in (Cortes et al., 2004). Although this method can easily achieve distributed coverage, it requires a large amount of computation, which costs too much when faced with a large area. Since then, a large number of researchers have extended the Voronoi diagram-based approach to solving more complex and practical coverage problems (Stergiopoulos and Tzes, 2009; Stergiopoulos and Tzes, 2010; Sun et al., 2019). Bagnitckii et al. (2017) used the AUV group to form a monitoring network and proposed a multi-objective optimal coverage control method for the AUV group based on a discrete information prediction method with known navigation trajectories. This method solves the problem of optimal coverage in the multi-target area of the AUV group, but its performance is insufficient in the face of unknown tracks and areas (Chen et al., 2021). Studied the optimal coverage control problem based on side-scan sonar, then designed an optimal navigation trajectory with less navigation consumption and wide monitoring area. This method has certain limitations on the load of the AUV. Similarly, to address the problem of prediction errors in the underwater environment, a least squares-based optimal coverage method was proposed in (Zhu et al., 2012). Wu et al. (2007) designed a multi-AUV distributed coverage control algorithm to make a homogeneous AUV group initially gathered together. This method is able to communicate with each other through the CVT method to be distributed in some specific pattern in a particular ocean area for scientific investigation. Although this method performs well on the optimal coverage of homogeneous AUV groups, it has certain limitations when facing HEAUVs.

It can be seen that the heterogeneous characteristics of the AUV group are rarely considered in the existing research of area coverage algorithms. When there is task load imbalance in HEAUVs, the CVT method cannot be directly used for static coverage control of the group. In order to solve the above problem, we first make a preliminary CVT division of the area according to the location optimal function, then combine the BCM to construct the allocation model of the target area. Finally, with the task demand capability ratio as the optimization goal, the uniform distribution of the target area of the heterogeneous AUV group is realized. The simulation results prove the superiority of the algorithm proposed in this paper. The main contributions of this paper are as follows:

1. A well-defined CVT method is provided for the AUV static area coverage problem. The target area can be segmented by this method based on location and sensitivity;
2. A biological competition mechanism model is designed to optimize the CVT distribution. AUV detection capabilities are linked to task requirements, which significantly improves the effectiveness of area allocation;
3. An area allocation optimization algorithm is proposed to achieve load balancing of task distribution while a strict proof of the consistency of the algorithm is provided based on the Lyapunov method;
4. Extensive simulation studies reveal the superior convergence of the proposed optimal static coverage control algorithm.

The rest of the paper is organized as follows. In Section 2, we state the static area coverage problem for HEAUVs. In Section 3, we design the optimal static coverage control algorithm based on BCM and prove the consistency of the algorithm. The simulation and results are described in Section 4. The conclusion is drawn in Section 5.

## 2 Problem Formulation

Due to the different manufacturers of each AUV, there are differences in the underlying technical architecture, equipment usage management, task load functions, which is called HEAUVs. In the case of harsh hydrological environment and complex detection tasks in the sea area, the highly intelligent and multifunctional cooperative detection of HEAUVs can complete tasks that cannot be completed or difficult to complete by single AUV and homogeneous AUV groups. When performing cooperative detection tasks, HEAUVs usually carry different equipment and resources, and the detection range and target type usually depend on the load capacity of each AUV. In addition, due to the unknown marine environment and underwater targets, it is more difficult to allocate the group detection area. Therefore, how to allocate the task detection area reasonably and efficiently is also a breakthrough and difficulty in developing a heterogeneous AUV group cooperative detection system.

Various types of AUVs usually carry different devices and resources, which causes a problem of unbalanced task load. Therefore, when HEAUVs are performing cooperative detection tasks, AUVs use the onboard sensing equipment to traverse all the positions of the detection target area through the coordination and cooperation mechanism to collect corresponding data. Among them, how to reasonably allocate the task area to make HEAUVs achieve the optimization goals of short task time, small repeated coverage area, low overall energy consumption and average task load is the focus of this paper.

When assigning task areas to HEAUVs, it is necessary to allocate the task areas reasonably according to the performance of each AUV. Based on the Voronoi area assignment and consistency conditions, the sub-detection areas of all AUVs are evenly distributed. In natural environments such as jungles, grasslands, and oceans, there is competition among organisms for resources such as living space and food, including interspecific competition and intraspecific competition. At the same time, it can also be found that, for any region, after a certain period of evolution, the competition among and within species can reach a state of relative equilibrium to achieve a reasonable allocation of resources. This result has many similarities with the task area allocation problem studied in this paper. Therefore, this paper will use a mathematical modeling of the above-mentioned biological competition mechanism (BCM), and analyze the theoretical basis that the model can realize the rational allocation of resources. On this basis, HEAUVs can reasonably allocate cooperative detection tasks only by simulating their dynamic processes.

Inspired by the resource ratio in BCM, let the number of targets appearing in the area be the task requirement *T*_*auv*_, the detection capability of each AUV is set with a constant *E*_*auv*_. The ratio of task requirements to detection capability can be defined as *R*_*auv*_ = *T*_*auv*_/*E*_*auv*_. The area forms the optimal allocation when and only when the *R*_*auv*_ of each AUV reaches consistency.

## 3 Coverage Algorithm Design

For the static area coverage problem of HEAUVs, the allocation scheme needs to be studied for each AUV to reach the designated area. In order to achieve the load balancing of task assignment, we propose a scheme to match the task requirements in the area with the detection capability of each AUV. Based on the CVT method, the scheme map the concept of resource ratio in BCM model to the ratio of task demand to detection capability in the area assignment. The overall algorithm steps are as follows.

**Algorithm 1:** Coverage Algorithm for HEAUVs. 
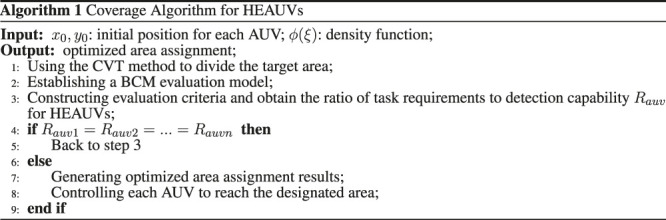


**FIGURE 1:**
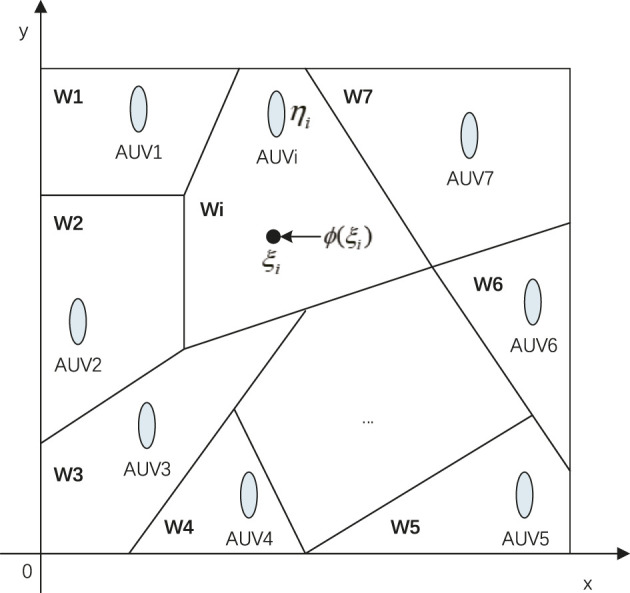
Schematic diagram of the area coverage problem.

We build the appropriate mathematical model based on the business problem requirements of static area coverage. Consider *n* AUVs randomly distributed in the target area $W\in R^{2}$ . The initial locations are $\eta =\left\{\eta_{1},\eta_{2},\ldots ,\eta_{n}\right\}$ , and *ξ* is any point in the area. The density function of the target area is $\phi \left(\xi \right):R^{2}\to R^{\;+\;}$ . Figure 1 shows a schematic diagram of the area coverage problem. Voronoi diagram method divides the target area into planar convex polygons.

In order to achieve CVT distribution in the target area, we take the position evaluation function as the optimization goal.

$$H\left(\eta ,W,\phi \right)=\overset{n}{\underset{i=1}{\sum }}\int_{W_{i}}f\left(\eta_{i},\xi \right)\phi \left(\xi \right)d\xi ,$$ (1)

where *W* = {*W*_1_, *W*_2_, … , *W*_*n*_} denotes the assigned area for each AUV, *f*(*η*_*i*_, *ξ*) denotes the measurement cost of the distance from the AUV to any point *ξ* in its assigned area. The purpose of this paper is to find the lowest *H*(*η*, *W*, *ϕ*) for each AUV in the assigned area, which is a distributed global optimization problem. The optimization process is based on global optimization.

### 3.1 CVT Distribution of the Target Area

In order to achieve static area coverage of the heterogeneous AUV group, the initial division of the target area needs to be completed first. And on this basis, a biological competition mechanism model is proposed for the secondary allocation of the target area.

The main methods for target area-oriented area partitioning are the raster method (Grediac et al., 2016), topological map (Ren et al., 2021), and Voronoi diagram (Cortes et al., 2004). The advantage of the raster method is that it is a method of representing the environment with approximate accuracy, good robustness, and good consistency in spatial expression. The disadvantage is that the raster update requires a large amount of calculation, which is not suitable for map representation in a large environment. The topological map is a compact map representation method, especially suitable for large and simple environments, but it loses applicability in the face of a complex sea environment. The Voronoi diagram generates several initial points in the plane randomly and connects two adjacent initial points to form a line segment, thereby forming a series of convex polygonal area divisions. The method is not affected by the size of the environment, and its applicability is broad.

In this paper, the Voronoi allocation principle is used to segment the target area. With Voronoi partitioning, the initial division of the target area can be achieved. The Voronoi division principle can be expressed as follow.

$$V_{i}=\left\{\xi \in W|f\left(\eta_{i},\xi \right)\leq f\left(\eta_{j},\xi \right),\forall i\neq j,\forall i,j\in n\right\},$$ (2)

where *V*_*i*_ denotes the Voronoi area of the *i*th AUV. An important property of this division area is that any point in the plane will be divided into the area in which the nearest initial point is located. However, the Voronoi division alone will make the area of each polygon vary in size, and the area is not uniform enough. During actual oceanographic tasks, we often want to make detailed observations of certain locations of interest. Therefore, the density function *ϕ*(*ξ*) is introduced to weight different locations in the ocean to describe the importance of the area. By defining the density function, the AUVs can gradually move toward the place where the density function is large, and finally form the desired stable distribution, which is centroid Voronoi distribution.

We assume that the group performs the coverage task at a fixed depth, so only the division within the two-dimensional area is considered in this paper. Define the location evaluation function of the *i*th AUV for its coverage area as Eq. 3.

$$H_{i}\left(\eta \right)=\int_{V_{i}}f\left(\eta_{i},\xi \right)\phi \left(\xi \right)d\xi ,$$ (3)

where *f*(*η*_*i*_, *ξ*) is the distance measurement function, defined as $f\left(\eta_{i},\xi \right)={\left\|\eta_{i}-\xi \right\|}^{2}$ , *ϕ*(*ξ*) is the sensitivity of random point *ξ* in the target area. Taking into account the location and sensitivity within the target area, the CVT distribution of the AUV group can be derived. We can observe that the CVT distribution is a distribution with the smallest position evaluation function. The characteristic of this distribution is that the points constituting the CVT distribution are both the initial point of the Voronoi diagram and the centroid of the Voronoi area. This results in a more uniform division of the entire area, and a certain distance is maintained between each initial point and its adjacent initial points. However, the CVT distribution does not consider the difference in individual detection capabilities of the heterogeneous AUV group. Although the result of the area division is uniform, the lack of adaptation to the detection capability of the AUVs can lead to the consequence that some AUVs are overburdened while others are underutilized. Therefore, the following will propose a biological competition mechanism model to further optimize the results of CVT distribution.

### 3.2 Biological Competition Mechanism Model

The process of biological competition involves many complex biological principles that cannot be precisely mathematically modeled (Wiens et al., 2014; Liu et al., 2022; Wu and Liu, 2022). This subsection focuses on the main features of AUV group coverage control, abstracting the process into a dynamic process determined by several key elements. Although the proposed model greatly simplifies the actual process, for the AUV group, the model contains the main system variables in the control. At the same time, abstract mathematical models make theoretical analysis possible and have stronger practicability. The definitions of the three elements of the model are given below, and the model is mapped accordingly with the coverage control of the heterogeneous AUV group.

• Power: indicates the competitiveness of individual organisms for resources. Power can indicate its ability to perform corresponding tasks, mainly determined by many factors such as AUV’s dynamic characteristics, sensor performance, and computing power.
• Territory: indicates the area controlled by an individual organism, for which all resources in the territory are occupied by the individual. In conjunction with the study in this paper, the territory can then denote the task area of each AUV.
• Resources: The total resources possessed by an organism, combined with the concept of territory, means that the total resources possessed by an organism are all the resources in its territory. Considering the threat probability model developed below, the concept of resources can be equated to the threat probability for each location in the area, with the total resources being the defined task load.

In addition, we assume that apart from the boundaries of territories, there is no overlap of territories and resource sharing among creatures. For coverage control, it means that an area does not need multiple AUVs to detect together. In the competition of nature, individuals at the upper level of the food chain often determine their possession of natural resources according to the degree of evolution. Combining the definitions of the above three elements, we can see that the greater the biological control, the more total resources they occupy. Therefore, this paper assumes a proportional relationship between the total resources of organisms and the control power, and proposes the following concept of resource ratio.

$$\text{Resource ratio }=\;\frac{Resource}{Power}.$$

According to the definition, the resource ratio represents the resources occupied by the unit’s control power. When there are several creatures in a certain area, and the resource ratio of each creature is not equal, encroachment will occur at the border of the territory. Creatures with higher resources will be invaded by creatures with lower resources. At the same time, the invaded creatures will adopt a strategy of retreat to avoid possible dangers, which will eventually lead to the reallocation of territory and resources. In this paper, the resource ratio corresponds to the task demand and detection capability ratio of AUVs. An evaluation model based on the biological competition mechanism is established to optimize the CVT method further.

### 3.3 Area Allocation Optimization Algorithm Based on Biological Competition Mechanism Model

In order to describe the correlation between the task area of AUV group and task requirements, we propose a position-probability model to describe the probability of the target appear in the search area. The larger the probability is, the wider the search area of the AUV group is, which means the larger the task payload of this AUV group. For any location *r* in a task region, assuming that there are *m* suspicious locations in the area. Target occurrence probability utilized by Gaussian probability function (Adeniran et al., 2020; Fritz, 2020) can be expressed as

$$target\left(r\right)=\overset{m}{\underset{i=1}{\sum }}\frac{1}{2\pi }\exp\left[-\frac{1}{2}{\left(r-r_{i}\right)}^{T}K_{i}\left(r-r_{i}\right)\right],$$ (4)

where *r*_*i*_ represents the location where the target is most likely to appear, which is mainly judged according to environmental features and prior knowledge. And we use random value in this work. *K*_*i*_ is a diagonal matrix, represents the probability weight of occurrence of *r*_*i*_. According to the above equation, the probability of occurrence of the target is a continuous function. Assuming that the location information of the *i*th AUV is *η*_*i*_, the task payload of the *i*th AUV can be expressed as the sum of the probability of occurrence of all targets in its task partition *V*.

$$task\left(i\right)=\underset{r\in V_{i}}{\int }target\left(r\right)dr.$$ (5)

Consider a convex polygon with *N* vertices as task area, then *V*_*i*_ is also a convex polygon. Let $\left\{v_{1},v_{2},\ldots ,v_{N}\right\}$ denotes the vertices, and the task area can be divided into *N* triangles whose vertices are respectively represented as (*η*_*i*_, *v*_*j*_, *v*_*j*+1_), where $j\in \left\{1,2,\ldots ,N-1\right\}$ . The task payload of the *i*th AUV can be expressed by a double integral:

$$task\left(i\right)=\overset{N}{\underset{j=1}{\sum }}\underset{s_{j}}{\int }\overset{m}{\underset{i=1}{\sum }}\frac{1}{2\pi }\exp\left[-\frac{1}{2}{\left(r-r_{i}\right)}^{T}K_{i}\left(r-r_{i}\right)\right]dr.$$ (6)

Assuming that the heterogeneous AUV group is randomly distributed in the task area, and the location is represented as $\eta =\left\{\eta_{1},\eta_{2},\ldots ,\eta_{n}\right\},\eta_{i}\in R^{2}$ . With the partitioning method defined above, we can get

$$V_{i}=\left\{\xi \in W|f\left(\eta_{i},\xi \right)\leq f\left(\eta_{j},\xi \right),\forall i\neq j,\forall i,j\in n\right\}.$$

The initial partition $\left\{V_{1},V_{2},\ldots ,V_{n}\right\}$ of all AUVs can be obtained, and the task payload of each AUV can be calculated by combining the target occurrence probability target(*r*), the sum of target occurrence probability *task*(*i*), which is represented as $\left\{T_{auv1},T_{auv2},\ldots ,T_{auvi}\right\}$ . We set a constant for the search capability of each AUV.

$$\left\{E_{auv1},E_{auv2},\ldots ,E_{auvi}\right\},$$ (7)

where *E*_*auvi*_ denotes the search capability of the *i* − th AUV.

The ratio of task and capability *R*_*auvi*_ = *T*_*auvi*_/*E*_*auvi*_ of each AUV can be calculated. Therefore, the consistency formula of the task payload can be represented as below:

$$R_{auv1}=R_{auv2}=\ldots =R_{auvi}.$$ (8)

*AUV*_*i*_ will move toward *AUV*_*j*_ when *R*_*auvi*_ < *R*_*auvj*_, and *i* ≠ *j*. Since the area assignment is based on the Voronoi partitioning principle, the task area of *AUV*_*j*_ will be reassigned to *AUV*_*i*_. The dynamics of the *i*th AUV is modelled as a second-order integral system:

$$\begin{array}{cc} & {\dot{p}}_{i}=\eta_{i}\\ & {\dot{\eta }}_{i}=u_{i}. \end{array}$$ (9)

The control input *u*_*i*_ of the system can be defined based on consistency theory and BCM (Bagnitckii et al., 2017):

$$u_{i}=-\gamma_{i}\overset{n}{\underset{j=1}{\sum }}{\vec{n}}_{ij}a_{ij}\left(\left(R_{auvi}-R_{auvj}\right)+b_{ij}\left(\eta_{auvi}-\eta_{auvj}\right)\right),$$ (10)

where *γ*_*i*_ is the feedback control gain coefficient greater than zero. ${\vec{n}}_{ij}=\frac{\eta_{i}-\eta_{j}}{\left\|\eta_{i}-\eta_{j}\right\|}$ is the direction vector. *a*_*ij*_ represents the degree of coupling between *AUV*_*i*_ and *AUV*_*j*_ in the task area. According to the occurrence probability of the target, *a*_*ij*_ can be obtained

$$a_{ij}=\underset{r\in V_{i}\cap V_{j}}{\int }target\left(r\right)dr.$$ (11)

### 3.4 Consistency Analysis

In order to prove whether the algorithm proposed in this paper can achieve the uniform distribution of the detection area, it is necessary to analyze its convergence.

Lemma 3.1. (Sayyaadi and Moarref, 2010) For n variables {δ_1_, δ_2_, … , δ_n_}, satisfy $\sum_{i=1}^{n}\delta_{i}=\Delta$ , where Δ is a constant value. Then for a set of positive real numbers {β_1_, β_2_, … , β_n_}, there exists:

$$\min\left(\sum_{i=1}^{n}\frac{\delta_{i}^{2}}{\beta_{i}}\right)=\frac{\Delta^{2}}{\sum_{i=1}^{n}\beta_{i}}.$$ (12)

And for $\left\{\delta_{1},\delta_{2},\ldots ,\delta_{n}\right\}$ , it satisfies

$$\frac{\delta_{i}}{\beta_{i}}=\frac{\Delta }{\sum_{i=1}^{n}\beta_{i}}.$$ (13)

Proof. According to the Lagrange multiplier method, the extremum of the objective function is obtained, and the Lagrange function is obtained as follows:

$$L\left(\delta_{1},\delta_{2},\ldots ,\delta_{n}\right)=\sum_{i=1}^{n}\frac{\delta_{i}^{2}}{\beta_{i}}+\lambda \left(\sum_{i=1}^{n}\delta_{i}-\Delta \right),$$ (14)

where *λ* is the Lagrange multiplier, then the extreme value of the function can be calculated by the following partial differential equation system

$$\frac{\partial L\left(\delta_{1},\delta_{2},\ldots ,\delta_{n}\right)}{\partial \delta_{i}}=0,\;i=1,2,\ldots ,n,$$ (15)

by Eq. 15, we can get

$$\frac{\delta_{1}}{\beta_{1}}=\frac{\delta_{2}}{\beta_{2}}=\cdots =\frac{\delta_{n}}{\beta_{n}},$$ (16)

by $\sum_{i=1}^{n}\delta_{i}=\Delta$ , the following conclusions can be drawn

$$\frac{\delta_{1}}{\beta_{1}}=\frac{\delta_{2}}{\beta_{2}}=\cdots =\frac{\delta_{n}}{\beta_{n}}=\frac{\Delta }{\sum_{i=1}^{n}\beta_{i}},$$ (17)

where

$$\sum_{i=1}^{n}\frac{\delta_{i}^{2}}{\beta_{i}}=\frac{\Delta^{2}}{\sum_{i=1}^{n}\beta_{i}}$$ (18)

Since the value in Eq. 18 is not a maximal value, Lemma 3.1 is proved.

According to Lemma 3.1, if the objective function can obtain a minimum value, the consistency shown in Eq. 17 can be achieved among the variables. Combining with the definition of resource ratio, it can be seen that the task payload *T*_*auvi*_ of *AUV*_*i*_ is taken as *δ*_*i*_, and the search capability *E*_*auvi*_ is taken as *β*_*i*_. When the search region does not change, there is the following formula:

$$T_{auv1}+T_{auv2}+\cdots +T_{auvn}=T_{S},$$ (19)

where *T*_*S*_ is a constant and represents the sum of the probability of targets occurrence in the search area. The conditions for the variable delta in the lemma can also be satisfied. Therefore, we can define a Lyapunov-like function as follows.

$$V=V_{1}+V_{2}+\cdots +V_{n}=\;\frac{T_{auv1}^{2}}{E_{auv1}}+\frac{T_{auv2}^{2}}{E_{auv2}}+\cdots +\frac{T_{auvn}^{2}}{E_{auvn}}.$$ (20)

Because the selected Lyapunov function cannot guarantee the inner $\dot{V}<0$ condition, the Lyapunov theory is subject to many limitations in the application process. The Lasalle invariance principle further generalizes the Lyapunov stability theory, and analyzes the asymptotic characteristics of the system by studying the position of the limit set of the dynamic system. In (Khalil, 2002), we can get the following definition of invariant set and Lasalle’s invariance theorem.

For a dynamic system whose definition domain is Ω ∈ *R* ^*n*^, if there is a set *M* ⊆Ω, and for any initial state $x\left(0\right)\in M$ of the system, all satisfy $x\left(t\right)\in M,\forall t\in R$ , then the set *M* is called the invariant set of the dynamic system. For a continuously differentiable function *V*, if in *M*, there is a set of system states when $\dot{V}\leq 0$ and *Q* is $\dot{V}=0$ , then for any initial state $x\left(0\right)\in M$ , the system state converges to the largest invariant set in *Q*.

Theorem 3.1. According to Lasalle’s invariance theorem, for a second-order integral system ${\dot{p}}_{i}=\eta_{i},{\dot{\eta }}_{i}=u_{i}$ with Eq. 10 as the system input. If and only if Lyapunov function achieves minimum value, the task-capability ratio of each AUV in the HEAUVs is consistent. That is, the search area is evenly distributed according to the load.

Proof. The time derivative of Eq. 20 can be expressed as

$$\begin{array}{cc} \frac{\partial V}{\partial t} & =\frac{\partial V_{1}}{\partial t}+\frac{\partial V_{2}}{\partial t}+\cdots +\frac{\partial V_{n}}{\partial t}\\ & =\left(\frac{\partial V_{1}}{\partial \xi }+\frac{\partial V_{2}}{\partial \xi }+\cdots +\frac{\partial V_{n}}{\partial \xi }\right)\frac{\partial \xi }{\partial t}\\ & =\overset{n}{\underset{i=1}{\sum }}\frac{\partial V}{\partial \eta_{i}}\frac{\partial \eta_{i}}{\partial t}, \end{array}$$ (21)

where $\frac{\partial \eta_{i}}{\partial t}=u_{i}$ . For *j* ≠ *i*, *j*∉*N*_*i*_ and *N*_*i*_ denotes the set of neighbors of the AUV*i*

$$\frac{\partial T_{auvi}}{\partial \eta_{j}}=0,$$ (22)

then

$$\frac{\partial V}{\partial \eta_{i}}=\frac{2T_{auvi}}{E_{auvi}}\frac{\partial T_{auvi}}{\partial \eta_{i}}+\underset{j\in N_{i}}{\sum }\frac{2T_{auvj}}{E_{auvj}}\frac{\partial T_{auvj}}{\partial \eta_{j}},$$ (23)

where *T*_*auvi*_ can be represented as

$$\begin{array}{cc} \frac{\partial T_{auvi}}{\partial \eta_{k}} & =\frac{\partial }{\partial \eta_{k}}\underset{r\in V_{i}}{\int }target\left(r\right)dr\\ & =\underset{r\in V_{i}}{\int }\frac{\partial }{\partial \eta_{k}}target\left(r\right)dr+\underset{\partial V_{i}}{\int }target\left(\mu \right)n^{T}\left(\mu \right)\frac{\partial \mu }{\partial \eta_{k}}d\mu , \end{array}$$ (24)

where *∂V*_*i*_ represents the boundary of *V*_*i*_, *μ* is the parameterized expression of the boundary. *n* ^*T*^(*μ*) is the outgoing normal line at the boundary, which is the unit vector. Since the probability distribution function target(*r*) of the occurrence of the target does not depend on the location of *η*_*k*_, $\underset{r\in V_{i}}{\int }\frac{\partial }{\partial \eta_{k}}target\left(r\right)dr$ is always zero. So we have the following formula

$$\frac{\partial T_{auvi}}{\partial \eta_{k}}=\underset{\partial V_{i}}{\int }target\left(\mu \right)n^{T}\left(\mu \right)\frac{\partial \mu }{\partial \eta_{k}}d\mu .$$ (25)

with $\partial V_{i}=\sum_{j\in N_{i}}V_{i}\cap V_{j}$ , the above equation can be expressed as

$$\frac{\partial T_{auvi}}{\partial \eta_{k}}=\sum_{j\in N_{i}}\underset{V_{i}\cap V_{j}}{\int }target\left(\mu_{ij}\right)n^{T}\left(\mu_{ij}\right)\frac{\partial \mu_{ij}}{\partial \eta_{k}}d\mu_{ij}.$$ (26)

In summary, we can get

$$\begin{array}{cc} \frac{\partial V}{\partial \eta_{i}} & =\frac{2T_{auvi}}{E_{auvi}}\underset{j\in N_{i}}{\sum }n^{T}\left(\mu_{ij}\right)\underset{V_{i}\cap V_{j}}{\int }target\left(\mu_{ij}\right)\frac{\partial \mu_{ij}}{\partial \eta_{i}}d\mu_{ij}\\ & +\underset{j\in N_{i}}{\sum }\frac{2T_{auvj}}{E_{auvj}}n^{T}\left(\mu_{ij}\right)\underset{V_{i}\cap V_{j}}{\int }target\left(\mu_{ji}\right)\frac{\partial \mu_{ji}}{\partial \eta_{i}}d\mu_{ji}. \end{array}$$ (27)

For any two adjacent *i* and *j*, *μ*_*ij*_ can be expressed as:

$$\mu_{ij}:\frac{\eta_{i}+\eta_{j}}{2}+\left[\begin{array}{cc} 0 & -1\\ 1 & 0 \end{array}\right]\frac{\eta_{i}-\eta_{j}}{\left\|\eta_{i}-\eta_{j}\right\|}\lambda_{ij},\lambda_{ij}\in \left[-a_{ij},b_{ij}\right].$$ (28)

where *μ*_*ij*_ is the perpendicular bisector of *η*_*i*_ and *η*_*j*_, *λ*_*ij*_ represents the boundary length. Since *μ*_*ij*_ and *n*(*μ*_*ij*_) are orthogonal, it can be concluded that:

$$n^{T}\left(\mu_{ij}\right)\frac{\partial \mu_{ij}}{\partial \eta_{i}}=n^{T}\left(\mu_{ij}\right)\frac{\partial \mu_{ij}}{\partial \eta_{j}}=\frac{1}{2}n^{T}\left(\mu_{ij}\right).$$ (29)

Substituted into Eq. 27, we can get:

$$\begin{array}{cc} \frac{\partial V}{\partial \eta_{i}} & =\frac{T_{auvi}}{E_{auvi}}\underset{j\in N_{i}}{\sum }n^{T}\left(\mu_{ij}\right)\underset{V_{i}\cap V_{j}}{\int }target\left(\mu_{ij}\right)d\mu_{ij}+\underset{j\in N_{i}}{\sum }\frac{T_{auvi}}{E_{auvi}}n^{T}\left(\mu_{ij}\right)\underset{V_{i}\cap V_{j}}{\int }target\left(\mu_{ji}\right)d\mu_{ji}\\ & +\underset{j\in N_{i}}{\sum }\frac{2T_{auvj}}{E_{auvj}}n^{T}\left(\mu_{ij}\right)\underset{V_{i}\cap V_{j}}{\int }target\left(\mu_{ji}\right)\frac{\partial \mu_{ji}}{\partial \eta_{i}}d\mu_{ji}. \end{array}$$ (30)

By further simplifying *n* ^*T*^(*μ*_*ij*_) = −*n* ^*T*^(*μ*_*ji*_)

$$\frac{\partial V}{\partial \eta_{i}}=\underset{j\in N_{i}}{\sum }\left(R_{auvi}-R_{auvj}\right)n^{T}\left(\mu_{ij}\right)\underset{V_{i}\cap V_{j}}{\int }target\left(\mu_{ji}\right)d\mu_{ji}.$$ (31)

then

$$\frac{\partial P}{\partial t}=-\overset{n}{\underset{i=1}{\sum }}k_{i}{\left\|\underset{j\in N_{i}}{\sum }\left(R_{auvi}-R_{auvj}\right)n\left(\mu_{ij}\right)a_{ij}\right\|}^{2}.$$ (32)

Since *V* is continuously differentiable and $\dot{V}\leq 0$ , according to the LaSalle invariance principle (Ding and Ding, 2016), if $\dot{V}=0$ , the system state values will converge to the maximum invariant set of the system. According to Eq. 32, when $\dot{V}=0$ ,

$$\underset{j\in N_{i}}{\sum }\left(R_{auvi}-R_{auvj}\right)n\left(\mu_{ij}\right)a_{ij}=0_{2\times 1}.$$ (33)

The matrix form of the above expression is expressed as:

$$\left(\begin{array}{ccc} l_{11} & \ldots & l_{1n}\\ \vdots & \ddots & \vdots \\ l_{m1} & \cdots & l_{mn} \end{array}\right)\left(\begin{array}{c} R_{auv1}\\ \vdots \\ R_{\text{auvn }} \end{array}\right)=\left(\begin{array}{c} 0_{2\times 1}\\ \vdots \\ 0_{2\times 1} \end{array}\right).$$ (34)

where $l_{ij}\in R^{2}$ , then

$$l_{ij}=\left\{\begin{array}{c} \underset{k\in N_{i}}{\sum }n\left(\mu_{ik}\right)a_{ik},j=i\;\\ -n\left(\mu_{ij}\right)a_{ij},j\in N_{i}.\; \end{array}\right.$$ (35)

Let $L_{\alpha }=L_{\alpha }^{U}⊗\left[\begin{array}{c} 1\\ 0 \end{array}\right]+L_{\alpha }^{D}⊗\left[\begin{array}{c} 0\\ 1 \end{array}\right]$ , where $L_{\alpha }^{U}$ , $L_{\alpha }^{D}$ represent the weighted Laplacian matrix of the system, and

$$\left\{\begin{array}{c} L_{\alpha }^{U}R_{auv}=0\;\\ L_{\alpha }^{U}R_{auv}=0.\; \end{array}\right.$$ (36)

where $R_{auv}={\left[\begin{array}{ccc} R_{auv1} & \cdots & R_{auvn} \end{array}\right]}^{T}$ is a column vector with the same elements, which is *R*_*auv*1_ = *R*_*auv*2_ = ⋯ = *R*_*auvn*_, then the theorem is proved.

## 4 Simulation Experiment and Analysis

### 4.1 Experimental Results

In order to verify the effectiveness of the method proposed in this paper, the following experiments were carried out. The simulation experiments are conducted on a PC with a 2.7 GHz Intel Core i7-5700HQ CPU (4 CPU cores) on a Windows 10 64-bit operating system. And python 3.7.10 is used to perform simulation experiments.

The area is set to 1,000 *m* × 1,000 *m*. The task area information is a prerequisite for performing the search task, and the task area is modeled with the threat probability as shown in Eq. 30. 10 suspicious target points are randomly generated within the area, and their horizontal and vertical coordinates are taken as integer values. For each suspicious point, the threat probability is chosen randomly between [0, 1]. The specific information is shown in Table 1. It is worth noting that, in the actual task, the suspicious target points are only obtained through analysis and judgment based on the regional situation and a priori knowledge.

**TABLE 1:** The location and threat probability of suspicious targets.

Suspicious target	Location	Threat probability
1	[729, 624]	0.534
2	[872, 410]	0.591
3	[467, 166]	0.810
4	[739, 574]	0.405
5	[425, 176]	0.761
6	[46, 409]	0.441
7	[487, 892]	0.467
8	[104, 432]	0.201
9	[488, 479]	0.935
10	[201, 573]	0.956

Based on the locations and the threat probabilities in Table 1, the probability of occurrence of the target for each coordinate is calculated using Eq. 37.

$$\text{target}\left(x,y\right)=\overset{4}{\underset{i=1}{\sum }}\text{targe}{\text{t}}_{i}\exp\left(-10^{-3}\left({\left(x_{i}-x\right)}^{2}+{\left(y_{i}-y\right)}^{2}\right)\right).$$ (37)

The task area is meshed with a precision of 1 m, and the task of each AUV is represented as the sum of the threat probabilities of all grid points in its task area. In this simulation, four HEAUVs are used to perform the coverage search task jointly. The task capability of each AUV is chosen within [0, 100]. The initial positions and the task execution capabilities are shown in Table 2.

**TABLE 2:** The initial location and the task execution capability of AUVs.

AUV	Initial location	Task execution capability
AUV1	[850, 542]	45.1
AUV2	[410, 327]	12.7
AUV3	[512, 4]	63.5
AUV4	[689, 132]	51.0

Combined with the information in Tables 1, 2, using the BCM-based area allocation optimization algorithm proposed in Section 3.3, the experimental results shown below can be obtained.

**FIGURE 2:**
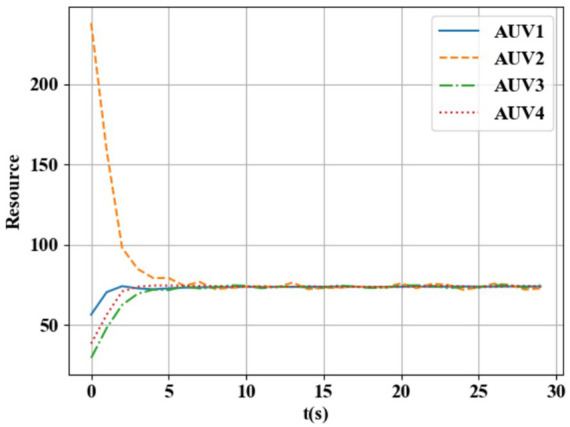
Resource ratio versus time curve.

**FIGURE 3:**
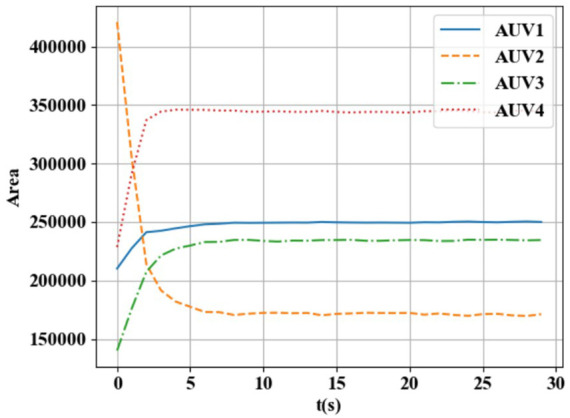
Area change curve with time.

From Figures 2, 3, it can be seen that the resource ratio of HEAUVs has achieved consistency, and its area has also achieved stability. Therefore, the experimental results validate the BCM-based region allocation algorithm proposed in Section 3.3.

### 4.2 Method Application

In this subsection, we apply the proposed method to perform the task of region assignment for HEAUVs. The initial scene settings remain the same as in Section 4.1. However, the initial positions and capabilities of HEAUVs vary, as shown in the Table 3.

**TABLE 3:** The initial location and the task execution capability of HEAUVs.

AUV	Initial location	Task execution capability
AUV1	[860, 552]	80.1
AUV2	[428, 527]	32.7
AUV3	[547, 1]	72.5
AUV4	[718, 97]	80.0
AUV5	[368, 687]	33.5
AUV6	[522, 91]	92.1
AUV7	[411, 837]	62.6

**FIGURE 4:**
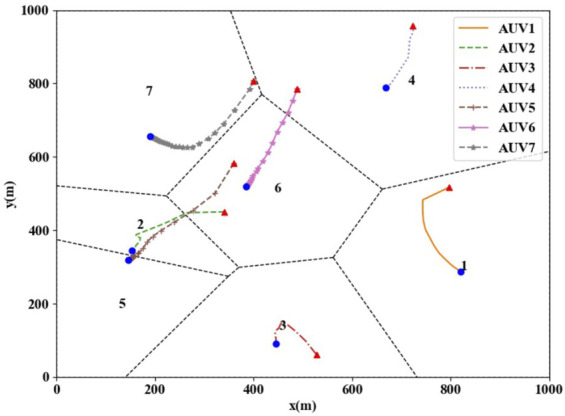
AUV group coverage trajectories.

**FIGURE 5:**
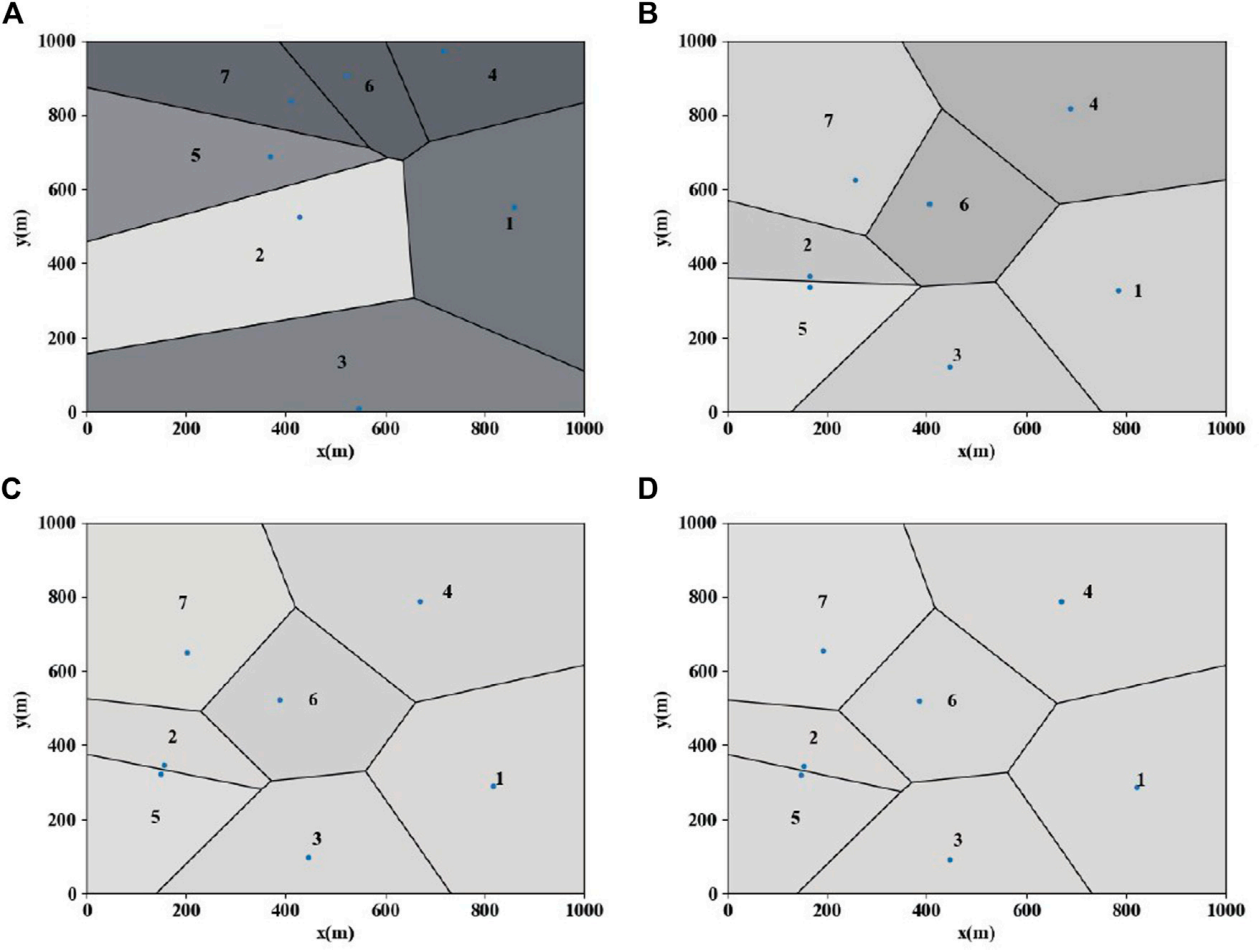
**(A)** Area allocation at time 10 s. **(B)** Area allocation at time 15 s. **(C)** Area allocation at time 20 s. **(D)** Area allocation at time 30 s.

After the initial CVT is generated, the state information is updated according to the optimal coverage control law in this paper until the task loads of all AUVs in the group are consistent. The simulation process is shown in Figures 4, 5. The triangle in Figure 4 indicates the initial positions of the AUV group coverage network, and the dot indicates the optimal coverage position. The navigational trajectories of the group are shown as the solid line, and the Voronoi diagram formed by the dashed line represents the optimal partition area of the AUV group coverage network. The simulation result shown in Figure 4 indicates that the AUV group coverage network can converge from any location in the area to the optimal coverage location under the control law presented in this paper.

**FIGURE 6:**
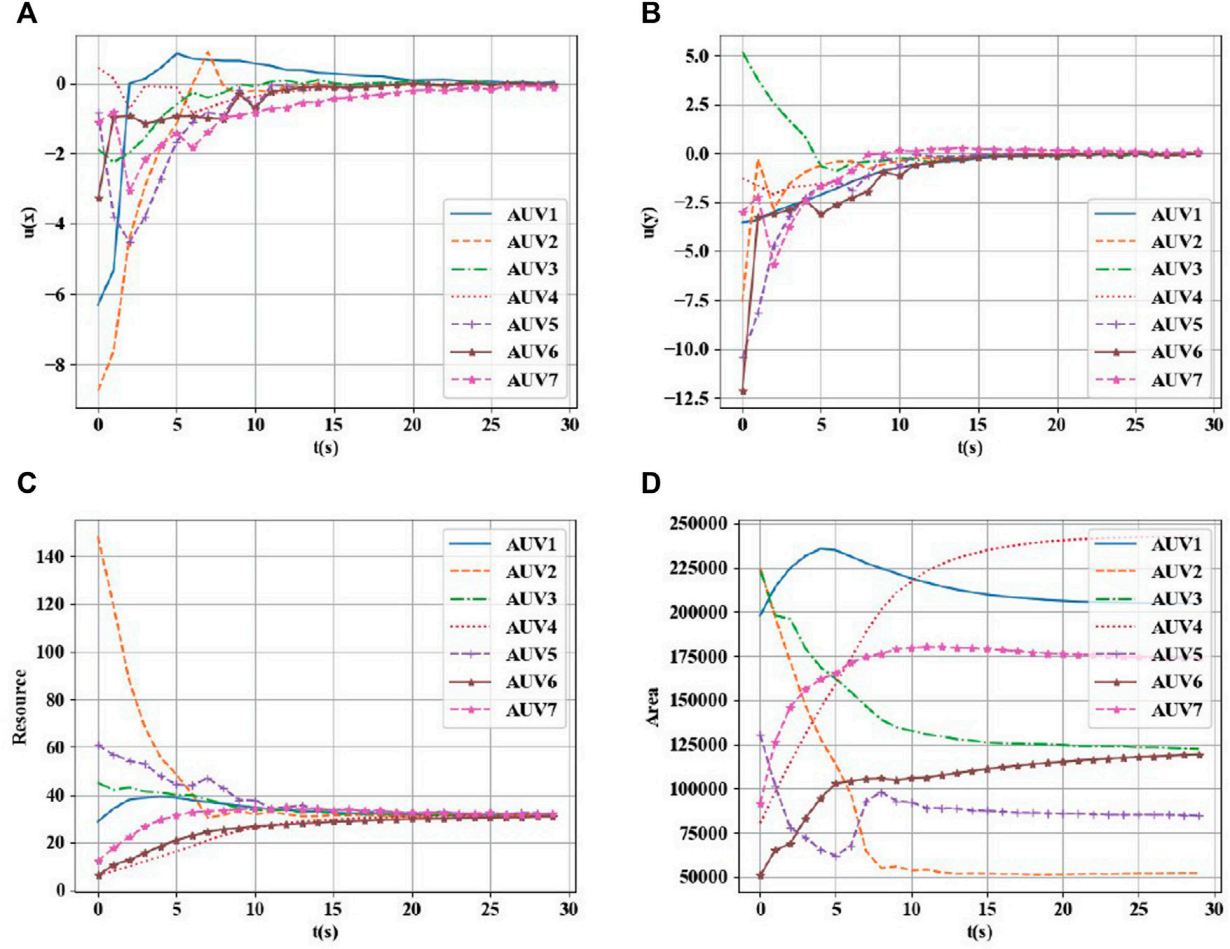
**(A)** *X* direction control value of AUVs. **(B)** *Y* direction control value of AUVs. **(C)** Total resource allocation. **(D)** Total area allocation.

The gray values in Figure 5 represent different target densities. The darker the color, the smaller the proportion. It can be seen from the final distribution map that the gray values of each area are the same. Based on the CVT optimal allocation, we successfully use the control law presented in this paper to achieve a balanced area allocation. The final state of the heterogeneous group is shown in Figure 6.

As shown in Figure 6, the method used in this paper can effectively achieve a balanced distribution of the given task area according to the task loads of different AUVs. The final task load ratio tends to be consistent, and the control values of each AUV in the *x* and *y* directions also converge to the same. The area values of the covered subregions also stabilized eventually.

### 4.3 Comparative Experiment

In order to demonstrate the advantages of the algorithm designed in this paper, two existing coverage control algorithms based on Voronoi partition (Lee et al., 2015; Miah et al., 2017) are used for simulation experiments to compare for a similar coverage control problem. The optimization objectives based on the biological competition model proposed in this paper are slightly different from those used in other literature. Therefore, two time integral-type evaluation metrics $H_{t}=\int_{0}^{t_{f}}H\left(\xi ,t\right)dt$ and $E_{t}=\int_{0}^{t_{f}}\overset{N}{\underset{i=1}{\sum }}\left\|e_{i}\left(t\right)\right\|dt$ are introduced for optimization performance comparison, where *H*_*t*_ is the integral of the optimization objective and $\left\|e_{i}\left(t\right)\right\|$ is the error between the AUV and its optimal position. The results of *H*_*t*_ show the convergence of the objective function, and the results of $\left\|e_{i}\left(t\right)\right\|$ indicate the convergence of tracking error. As shown in Table 4, the method proposed in this paper performs better than the other two methods. The values of both evaluation indexes are minimum.

**TABLE 4:** Performance comparison of different coverage control algorithms.

Evaluation index	This paper	Reference (Lee et al., 2015)	Reference (Miah et al., 2017)
$H_{t}=\int_{0}^{t_{f}}H\left(\xi ,t\right)dt$	134.226	135.537	135.238
$E_{t}=\int_{0}^{t_{f}}\overset{N}{\underset{i=1}{\sum }}\left\|e_{i}\left(t\right)\right\|dt$	3141.45	3161.13	3165.62

Several more scenarios are selected for numerical comparison and validation of the proposed method with (Lee et al., 2015; Miah et al., 2017). The area range is set to 1,000 *m* × 1,000 *m* and 2,000 *m* × 2,000 *m* respectively. The experiment is conducted using groups of 10/20/30/40/50 AUVs. The initial position and task execution capability of each AUV are chosen randomly, with the initial position within the set area and the capability value in the range [10, 100]. The number of threat targets uses random values in the range [10, 20] for an area size of 1,000 *m* × 1,000 *m* and [20, 40] for an area size of 2,000 *m* × 2,000 *m*, with threat values in the range [0.1, 1.0].

A total of 6 groups of tests are conducted, where each test is conducted 100 times, and the mean and variance of the performance evaluation indexes are calculated. In each case, the AUVs are initialized to the same positions to mitigate an inherently problematic comparison. As shown in Tables 5, 6. In all cases the proposed method performs better than (Lee et al., 2015; Miah et al., 2017), which is because the BCM ensures the speed of area allocation. The percent reduction comparing the proposed method and the other methods is shown in Table 7, indicating the superiority of the proposed method. Moreover, the proposed method guarantees load balancing under different AUV task execution capability, which is not considered by the compared algorithms.

**TABLE 5:** Comparison of algorithms performance under 1,000 *m* × 1,000 *m* area range.

Number of AUVs	This paper				Reference (Lee et al., 2015)				Reference (Miah et al., 2017)			
	*H*_*t*_		*E*_*t*_		*H*_*t*_		*E*_*t*_		*H*_*t*_		*E*_*t*_	
	Mean	Variance	Mean	Variance	Mean	Variance	Mean	Variance	Mean	Variance	Mean	Variance
10	158.16	16.21	3325.34	89.12	167.59	15.30	3371.55	86.02	163.48	16.05	3378.16	90.19
20	182.35	18.11	3712.72	97.32	189.49	17.41	3777.12	96.94	185.84	17.59	3784.28	92.61
30	197.85	19.62	4026.11	91.39	204.83	19.46	4051.83	85.26	199.42	21.25	4055.69	88.21
40	224.81	18.62	4366.41	101.44	229.15	19.84	4371.47	99.88	226.23	20.73	4382.37	94.56
50	241.13	20.77	4621.83	96.79	248.51	21.04	4683.19	97.92	245.81	20.24	4690.26	98.49

**TABLE 6:** Comparison of algorithms performance under 2,000 *m* × 2,000 *m* area range.

Number of AUVs	This paper				Reference (Lee et al., 2015)				Reference (Miah et al., 2017)			
	*H*_*t*_		*E*_*t*_		*H*_*t*_		*E*_*t*_		*H*_*t*_		*E*_*t*_	
	Mean	Variance	Mean	Variance	Mean	Variance	Mean	Variance	Mean	Variance	Mean	Variance
10	718.84	27.89	10208.30	194.67	739.59	27.80	10312.38	196.30	727.94	28.21	10337.47	198.90
20	813.19	29.75	11609.51	198.61	835.97	30.05	11698.52	199.76	824.56	29.22	11716.51	195.40
30	852.23	29.80	12280.23	203.72	889.75	28.77	12394.93	202.49	866.15	29.56	12409.64	203.61
40	971.07	33.79	13135.86	195.09	1020.43	31.76	13229.71	196.96	1003.80	31.71	13248.87	198.72
50	1091.09	31.40	14357.12	197.34	1118.67	32.59	14461.13	204.26	1112.85	30.15	14483.26	202.76

**TABLE 7:** Percent reduction in the mean value of evaluation metrics between the proposed method and the comparison methods.

Area range	Number of AUVs	Reference (Lee et al., 2015)		Reference (Miah et al., 2017)	
		*H*_*t*_	*E*_*t*_	*H*_*t*_	*E*_*t*_
1,000 *m* × 1,000 *m*	10	−5.96%	−1.39%	−3.36%	−1.59%
	20	−3.92%	−1.73%	−1.91%	−1.93%
	30	−3.53%	−0.64%	−0.79%	−0.73%
	40	−1.93%	−0.12%	−0.63%	−0.37%
	50	−3.06%	−1.33%	−1.94%	−1.48%
2,000 *m* × 2,000 *m*	10	−2.81%	−1.02%	−1.25%	−1.27%
	20	−2.72%	−0.77%	−1.38%	−0.92%
	30	−4.22%	−0.93%	−1.61%	−1.05%
	40	−2.67%	−0.71%	−2.06%	−0.86%
	50	−2.47%	−0.72%	−1.96%	−0.88%

## 5 Conclusion

A novel algorithm for static area coverage control of the heterogeneous AUV group in an unknown environment has been introduced in this study. First, the initial partitioning of the target area based on the CVT method is given. Then, a dynamic model of the regional allocation of the heterogeneous AUV group based on the biological competition mechanism is developed. Finally, the task demand to capacity ratio is used as an evaluation criterion to achieve a uniform and consistent task load. The consistency of the algorithm is proved based on the Lyapunov method. The stability and superiority of the area coverage algorithm proposed in this paper are verified by comparison with simulation experiments.

The static area coverage algorithm of HEAUVs proposed in this paper does not consider the situation that there are dynamic or static obstacles in the target area. When there are dynamically changing obstacles in the area, we also need to consider the problem that the AUV avoids obstacles in the process of traveling. This is a complex multi-objective optimization problem that needs our further study. At the same time, the movement of the target was not taken into account under the disturbance of wind, waves and currents on the sea surface. The different anti-interference capabilities of each AUV will also affect the results of area allocation, which is also a constraint that needs to be considered. Therefore, in future research work, we can consider studying the dynamic area coverage of HEAUVs under the disturbance of marine environment.

The research in this paper can be applied to HEAVUS’s collaborative reconnaissance and early warning, on-call submarine search, and swarm formation operations, and it also has good compatibility with homogeneous AUV groups. However, the research of the algorithm in this paper is still in the simulation experiment, and needs to be further verified in the dynamic changing real sea area.

## Data Availability

The original contributions presented in the study are included in the article/Supplementary Material, further inquiries can be directed to the corresponding author.
